# Atrial Substrate Modification in Atrial Fibrillation: Targeting GP or CFAE? Evidence from Meta-Analysis of Clinical Trials

**DOI:** 10.1371/journal.pone.0164989

**Published:** 2016-10-20

**Authors:** Mu Qin, Xu Liu, Shao-Hui Wu, Xiao-Dong Zhang

**Affiliations:** Department of Cardiology, Shanghai Chest Hospital Affiliated to Shanghai Jiaotong University, Shanghai 200030, China; University of Minnesota, UNITED STATES

## Abstract

Several clinically relevant outcomes post atrial substrate modification in patients with atrial fibrillation (AF) have not been systematically analyzed among published studies on adjunctive cardiac ganglionated plexi (GP) or complex fractionated atrial electograms (CFAE) ablation vs. pulmonary vein isolation (PVI) alone. Out of 176 reports identified, the present meta-analysis included 14 randomized and non-randomized controlled trials (1613 patients) meeting inclusion criteria. Addition of GP ablation to PVI significantly increased freedom from atrial tachyarrhythmia in short- (OR: 1.72; P = 0.003) and long-term (OR: 2.0, P = 0.0006) follow-up, while adjunctive CFAE ablation did not after one or repeat procedure (P<0.05). The percentage of atrial tachycardia or atrial flutter (AT/AFL) after one procedure was higher for CFAE than GP ablation. In sub-analysis of non-paroxysmal AF, relative to PVI alone, adjunctive GP but not CFAE ablation significantly increased sinus rhythm maintenance (OR: 1.88, P = 0.01; and OR:1.24, P = 0.18, respectively). Meta regression analysis of the 14 studies indicated that sample size was significant source of heterogeneity either in outcomes after one or repeat procedure. In conclusion, in patients with AF, adjunctive GP but not CFAE ablation appeared to significantly add to the beneficial effects on sinus rhythm maintenance of PVI ablation alone; and CFAE ablation was associated with higher incidence of subsequent AT/AFL.

## Introduction

Catheter ablation for persistent atrial fibrillation (AF) is more challenging and yields less favorable outcomes. To improve outcomes, ablation targeting the left atrial substrate that maintains fibrillation is often added to pulmonary vein isolation (PVI), with ganglionated plexi (GP) and complex fractionated atrial electrograms (CFAE) ablation respectively targeting major GPs around pulmonary veins and complex atrial signals. Clinical and experimental studies suggest a link between GP and CFAE: acetylcholine-induced activation of cardiac GP provokes CFAE[1], and CFAE distribution follows that of areas in the left atrium and pulmonary veins that are richly innervated by cardiac autonomic nerves[2].

However, GP/CFAE ablation has yielded inconsistent results. Among patients with persistent and paroxysmal AF, Scherlag et al showed that GP ablation in addition to PVI increased ablation success from 70% to 91% at 12 months follow-up[3], while Pokushalov et al found that only 50% of patients were free from recurrent AF after undergoing combined GP ablation and PVI[4,5]. A meta-analysis had advocated that as adjunctive strategy, CFAE ablation was associated with encouraging results[6] while in several studies, it did not reduce rate of recurrent atrial fibrillation when compared with PVI alone[7–9]. Although pooled analyses have evaluated the effectiveness of the GP/CFAE strategy, several clinically relevant data such as long-term outcomes (>1 year), success rates after one or multiple procedures, and tachyarrhythmia recurrence type remain unclear. We therefore here further evaluated the efficacy of adjunctive GP and CFAE ablation strategies by systematic review of randomized clinical trials (RCTs) and non-RCTs.

## Methods

### Database search

The key terms “atrial fibrillation,” “GP ablation,” “CFAE ablation,” and “pulmonary vein isolation” were used to systematically search PubMed, Elsevier, the Cochrane Library, and the China National Knowledge Infrastructure (CNKI) from 2004 to the end of 2014. In addition, the abstracts of conferences and references of the identified papers and reviews were examined. The following predefined exclusion criteria were used: 1) non-controlled trials; 2) no mention of original data on AF elimination; 3) study neither compared CFAE ablation plus PVI with PVI nor GP ablation plus PVI with PVI; and 4) follow-up duration was <6 months.

### Data extraction

All literature searches were reviewed independently by two of the authors (Mu Qin and Shao-hui Wu), and results were recorded on a standardized data extraction form. Disagreements were resolved by consensus.

### Statistical analysis

All continuous variables are presented as mean±standard deviation. Categorical data are summarized as frequencies and percentages. Odds ratios (ORs) with 95% confidence intervals (CIs) were estimated by using random effects models (REM) or fixed effects models (FEM) based on the individual ORs. Heterogeneity between studies was calculated by using the Chi-square test and I^2^ score, with a higher I^2^ score denoting greater heterogeneity. If the p-value for heterogeneity was >0.1 or I^2^ was <50%, the FEM was used; otherwise, the REM was chosen. All p-values were two-tailed, and p<0.05 was considered to indicate statistical significance. The meta-analysis data were analyzed using RevMan 5.0 software. Statistical analyses were performed with REVMAN software (version 5.2; Cochrane Collaboration, Oxford, United Kingdom). We used STATA 13.0 to perform meta-regression for assessment of the source of heterogeneity.

## Results

### Search results

**Fig 1:**
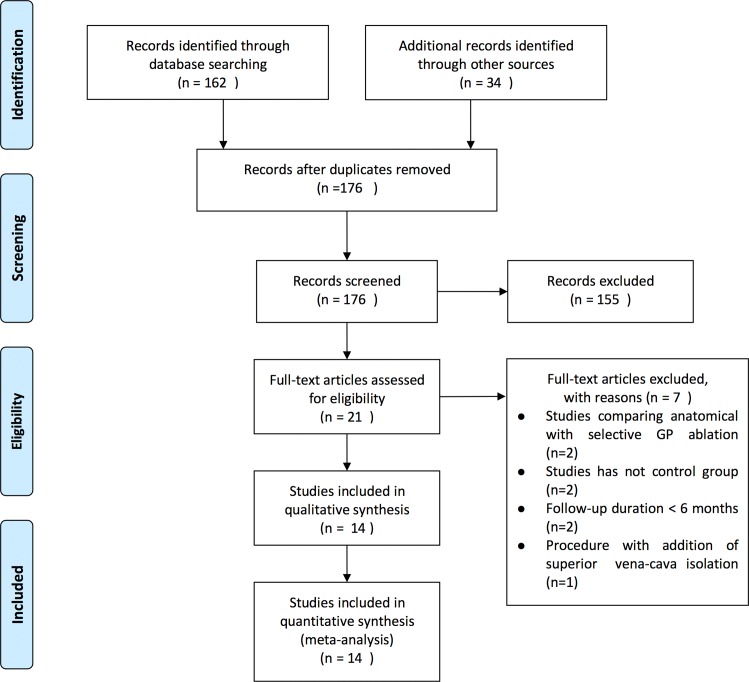
Results of literature search for the meta-analysis

In total, 176 relevant articles were retrieved from MEDLINE, EMBASE, and CCRT; 21 clinical trials that fulfilled the eligibility criteria were identified. Among these manuscripts, seven trials were excluded from the analysis for the following reasons: two because the study compared anatomical with selective GP ablation; one focused on long-term success rate of anatomic GP ablation in chronic AF; one recorded autonomic GP responses during PVI; two only compared GP ablation alone with PVI; two had short-term follow-up; and another added superior vena cava isolation (Fig 1). Therefore, after full manuscript review, 14 trials were included for analysis[3,5,7–18], and 5 had three treatment groups [7,10,14,15,17]. Of note, the investigators Katritsis and Verma were lead authors on multiple included trials; however, the subjects in these trials did not overlap, and 3 were multicenter studies[7,10,17].

### Study characteristics

The basic features of the trials, including procedural details and primary endpoints, are presented in Table 1. Nine of the 14 eligible trials were RCTs. Definition of recurrence differed slightly among included trials. In Pokushalov E et al, patients with an AF% >0.5 were classified as experiencing AF recurrence; the 0.5% cutoff corresponded to a maximum cumulative time in AF of 3.6 hours at 1 month and to >99.5% of the time spent in sinus rhythm during the overall follow-up period. The other studies considered AF recurrence as AF or atrial arrhythmia lasting for more than 30 or 60 seconds. Anti-arrhythmic drugs in all trials were discontinued within 2–3 months after ablation except in one, in which 10–15% patients were on them until the end of the trial.

**Table 1:** Detailed procedures of included trials.

Trials	Treatment	Design	Follow-up (month)	Ablation Area	Target Identification	AADs Post- procedure	Primary Endpoint
***GP Ablation Strategy***							
Scherlag 2005 ^3^	GP+PVI vs PVI	CCT	1–15	SLGP, ILGP, ARGP, IRGP	Selective	___	Freedom from AF
Pokushalov 2013 ^5^	GP+PVI vs PVI+LL	RCT	36	SLGP, ILGP, ARGP, IRGP, MGP	Selective	3 months	Freedom from AF (AF burden≤0.5%)
Katritsis 2013 ^10^	GP+PVI vs PVI	RCT	24	SLGP, ILGP, ARGP, IRGP	Anatomic	3 months	Freedom from AF or other sustained (duration>30s) atrial tachyarrhythmia
Katritsis 2011 ^11^	GP+PVI vs PVI	RCT	11.3±1.9	SLGP, ILGP, ARGP, IRGP	Anatomic	2 months	Freedom from AF or other sustained (duration>30s) atrial tachyarrhythmia
***CFAE Ablation Strategy***							
Verma 2015 ^7^	CFAE+PVI vs PVI	RCT	18	LA+RA+CS	Automated	3 months	Freedom from AF or other sustained (duration>30s) atrial tachyarrhythmia
Vogler 2015 ^8^	CFAE+PVI vs PVI	RCT	12	LA+RA+CS	Automated	3 months	No AF or other atrial arrhythmia on Holter monitor
Oral 2009 ^9^	CFAE+PVI vs PVI	RCT	10±3	LA+CS	Automated	8–12 weeks	Freedom from AF or other sustained (duration>30s) atrial tachyarrhythmia 12 weeks after procedure
Verma 2008 ^12^	CFAE+PVI vs PVI	CCT	12–18	LA+CS	Automated	2 months	No AF or other atrial arrhythmia on ECG/Holter monitor 3 months after procedure
Lin 2009 ^13^	CFAE+PVI+LL vs PVI+LL	CCT	19±11	LA+CS	Automated	8 weeks	Freedom from AF or other sustained (duration>1min) atrial
Chen 2011 ^14^	CFAE+PVI vs PVI	RCT	22.6±6.4	LA+CS	Automated	No more	No AF or other sustained atrial arrhythmia (duration>1min) on ECG/Holter monitor 3 months after procedure without AADs
Baise 2009 ^15^	CFAE+PVI vs PVI	RCT	13.7±2.2	LA+RA+CS	Automated	2 months	No episodes of AF/AT (duration>1min) 2 months after procedure with or without AADs
Elayi 2008 ^16^	CFAE+PVI vs PVI	RCT	16±1	LA+RA+CS	Automated	2 months	Freedom from AF or other sustained (duration>1min) atrial tachyarrhythmia 2 months after procedure without AADs
Verma 2010 ^17^	CFA E+PVI vs PVI	RCT	12	LA+RA+CS	Automated	2 months	Freedom from AF or other sustained (duration>30s) atrial tachyarrhythmia 3 months after procedure
Nam 2012 ^18^	CFAE+PVI vs PVI	CCT	23±12	LA+RA+CS	Automated	Reinitiated if symptomatic recurred	No episodes of AF/AFL on ECG/Holter monitor 3 months after procedure without AADs

AF, atrial fibrillation; GP, ganglionated plexi; PVI, pulmonary vein isolation; RCT, randomized controlled trial; CCT, controlled clinical trial; SLGP, superior left GP; ILGP, inferior left GP; ARGP, anterior right GP; IRGP inferior right GP; MGP, Mashall tract GP; HFS, high frequency stimulation; AADs, anti-arrhythmia drugs.

Table 2 summarizes the baseline characteristics of enrolled patients. The 14 studies included encompassed a total of 1613 patients: 575 (35.6%) with paroxysmal AF and 1038 (64.3%) with non-paroxysmal AF; and 673 (41.7%) underwent GP ablation, and 940 (58.3%) underwent CFAE ablation. Mean age, left atrial dimension and left ventricular ejection fraction were not significantly different between experimental and control groups in each included trial.

**Table 2:** Characteristics of patients in each ablation strategy.

Trails	No of Patients		Age (y)		Male (%)		PAF (%)		LVEF (%)		LA Diameter (mm)	
	E	C	E	C	E	C	E	C	E	C	E	C
***Adjunctive GP Ablation Strategy***												
Scherlag 2005	33	27	—	—	—	—	51	52	—	—	—	—
Pokushalov 2013	132	132	55±6	54±7	76	79	0	0	55.1±4.8	54.2±6.3	49±7	48±7
Katritsis 2011	34	33	55.2±11.6	53.2±11.3	73	79	100	100	56.2±7.7	56.1±5.3	41.5±5.4	41.1±3.3
Katritsis 2013	82	78	56±8.5	56±7.6	69	68	100	100	62±8.1	63±6.8	48±6	48±7
***Adjunctive CFAE Ablation Strategy***												
Verma 2015	244	61	58±10	60±9	52	213	0	0	57±10	55±11	44±6	44±6
Vogler 2015	71	61	61.1± 10.9	63.0± 9.6	60	56	0	0	59.8 ± 7.1	60.0 ± 7.1	43.7± 5.2	44.5 ± 6.6
Oral 2009	50	50	62±8	58±10	82	82	0	0	54±9	53±12	46±6	47±6
Verma 2008	35	35	61±9	60±11	74	77	60	60	53±7	53±8	43±9	41±10
Lin 2009	30	30	49±10	49±12	80	87	0	0	54±8	56±8	41±7.6	40±4.7
Chen 2011	58	35	56.4±11.2	52.2±13.2	67	71	100	100	64.5±3.3	66.2±4.1	34.2±3.6	34.7±4.2
Baise 2009	34	35	58.4±7.5	57±8.1	88	83	100	100	54.6±6	55±8	44±6	43±6
Elayi 2008	49	48	59.2±11.5	58.1±10.3	65	69	0	0	55	52	46.2±6.4	45.1±6.6
Verma 2010	34	32	59±10	55±11	74	75	65	66	59±12	62±7	41±6	43±5
Nam 2012	35	35	54±11	55±11	86	86	100	100	59±6	61±5	40±5.1	40±4.5

E, experiment group (GP+PVI); C, control group (PVI); PAF, paroxysmal AF; LVEF, left ventricular ejection fraction; LA, left atria.

### Atrial tachyarrhythmia recurrence after one procedure

**Fig 2:**
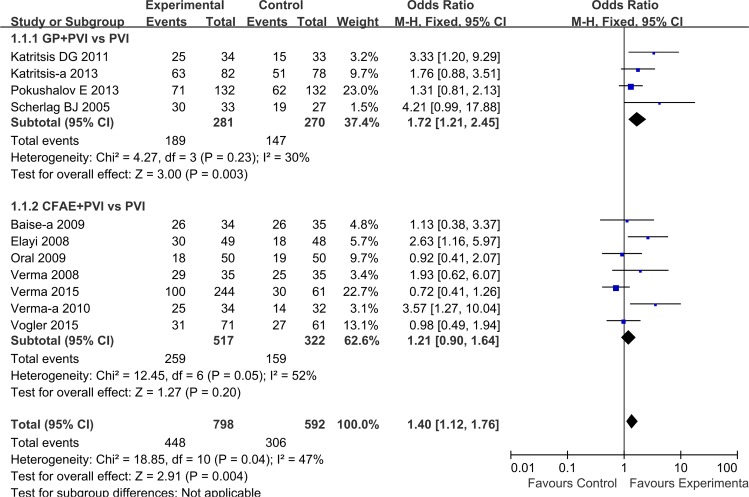
Effects of additional GP/CFAE ablation on short-term sinus rhythm maintenance after one procedure.

**Fig 3:**
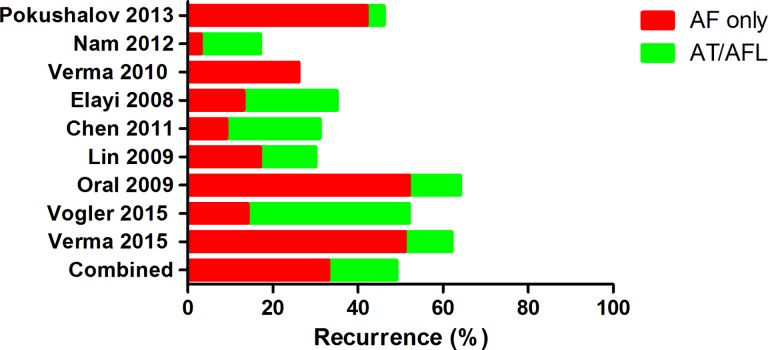
Type of atrial tachyarrhythmia recurrence by ablation strategy. AF, atrial fibrillation; AT, atrial tachycardia; AFL, atrial flutter.

For all studies combined, there was significant benefit to the addition of GP or CFAE ablation to PVI in terms of freedom from AF at short-term follow-up (OR: 1.40, 95%CI: [1.12, 1.76]; P = 0.004). Heterogeneity among studies was significant (I^2^ = 47%, P = 0.04) (Fig 2). In subgroup analysis, addition of GP ablation increased rates of freedom from atrial tachyarrhythmia (1.72 [1.21, 2.45]; P = 0.003), and only one trial reported atrial tachyarrhythmia recurrence type: 42% and 4% for AF and AT/AFL, respectively (Fig 3 and Table 3). However, in analysis of CFAE+PVI strategy versus PVI alone, seven studies showed similar rate of sinus rhythm maintenance, 50.1% vs 49.4% (OR = 1.21 [0.90, 1.64]; P = 0.20) (Fig 2). Moreover, as per data from six studies, 36% of patients experienced AF-only recurrence, and 16% had AT or AFL at short-term follow-up (Fig 3 and Table 3), with the difference between two groups being not significant (P>0.05). Notably, bi-atrial CFAE ablation was less efficacious than ablation at LA only, with respective arrhythmia freedom rates of 49% and 55% at 1-year follow-up.

**Table 3:** Atrial tachyarrhythmia recurrence in each ablation strategy.

Trails	No. of Patients		Freedom after One Procedure(%)		AF Recurrence after One Procedure (%)		AT/AFL Recurrence after One Procedure (%)		No. of Patients with Repeat Procedure (%)		Freedom after Repeat Procedure(%)	
	E	C	E	C	E	C	E	C	E	C	E	C
***Adjunctive GP Ablation Strategy***												
Scherlag 2005	33	27	30 (91)	19 (70)	—	—	—	—	—	—	—	—
Pokushalov 2013	132	132	65 (49)	45 (34)	56 (42)	50 (38)	5 (4)	20 (15)	55 (42)	78 (59)	90 (68)	69 (52)
Katritsis 2011	34	33	25 (74)	15 (45)	—	—	—	—	6 (18)	7 (21)	29 (85)	20 (61)
Katritsis 2013	82	78	61 (74)	44 (56)	—	—	—	—	—	—	—	—
***Adjunctive CFAE Ablation Strategy***												
Verma 2015	244	61	100 (41)	30 (49)	125(51)	25(57)	27 (11)	7 (11)	63 (26)	13 (21)	122 (50)	37 (61)
Vogler 2015	71	61	31(44)	27(44)	10(14)	9(15)	27(38)	18(29)	29(41)	25(41)	36(51)	37(61)
Oral 2009	50	50	18 (30)	19 (38)	26 (52)	29 (58)	6 (12)	2 (4)	17 (34)	18 (36)	30 (60)	34 (68)
Verma 2008	35	35	29 (83)	25 (71)	—	—	—	—	—	—	—	—
Lin 2009	30	30	20 (67)	11 (37)	5 (17)	15 (50)	4 (13)	3 (10)	5 (17)	13 (43)	23 (77)	18 (60)
Chen 2011	58	35	40 (69)	27 (77)	5 (9)	6 (17)	13 (22)	2 (6)	—	—	—	—
Baise 2009	34	35	26 (76)	26 (74)	—	—	—	—	4 (12)	3 (9)	29 (85)	29 (83)
Elayi 2008	49	48	30 (61)	18 (37)	8 (13)	15 (31)	11 (22)	14 (29)	10 (20)	12 (27)	39 (80)	27 (56)
Verma 2010	34	32	25 (73)	14 (44)	9 (26)	17 (53)	0 (0)	1 (3)	5 (15)	10 (31)	30 (88)	22 (69)
Nam 2012	35	35	29 (83)	22 (63)	1 (3)	13 (37)	5 (14)	0 (0)	3 (9)	2 (6)	31 (89)	24 (69)

E, experiment group (GP+PVI); C, control group (PVI).

### Long-term success rate after one procedure

**Fig 4:**
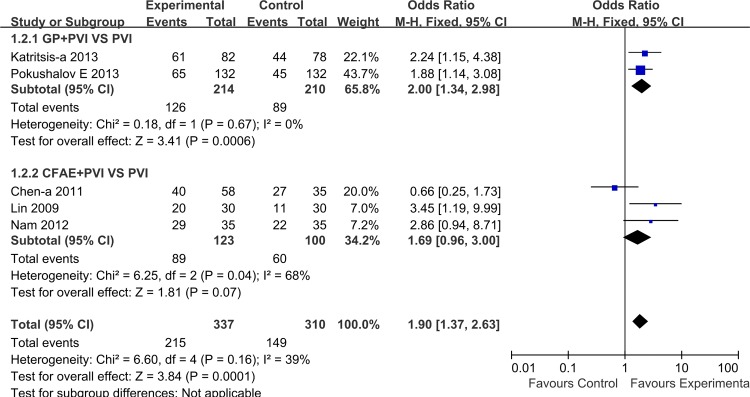
Effects of additional GP/CFAE ablation on long-term sinus rhythm maintenance after one procedure.

Five studies, encompassing 364 patients, reported long-term results for primary outcomes with two also reporting short-term follow-up results. Overall, the pooled estimate showed that compared to PVI, adjunctive GP/CFAE ablation further increased long-term sinus rhythm maintenance (1.90 [1.37, 2.63]; P = 0.0001), without significant heterogeneity among included studies (I^2^ = 39%, P = 0.16). Subgroup analysis showed better sinus rhythm maintenance in favor of GP/CFAE plus PVI, with the difference being statistically significant only for adjunctive GP ablation (2.0 [1.34, 2.98]; P = 0.0006) (Fig 4).

### Comparison of efficacy between PAF and non-PAF

**Fig 5:**
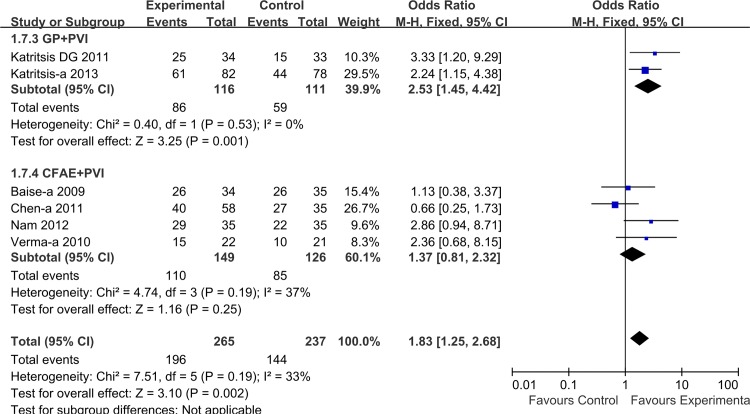
Effects of additional GP/CFAE ablation on short-term sinus rhythm maintenance in patients with paroxysmal AF.

**Fig 6:**
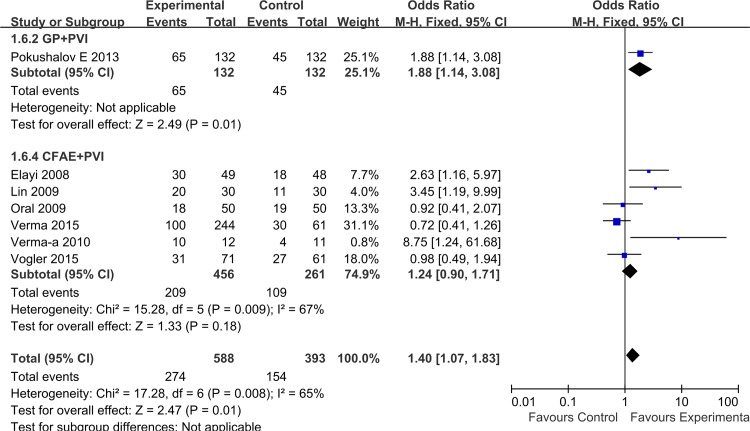
Effects of additional GP/CFAE ablation on short-term sinus rhythm maintenance in patients with non-paroxysmal AF.

To assess the impact of AF characteristics on overall treatment effect, we performed a separate analysis after classification of AF into paroxysmal and non-paroxysmal AF. Combining the six trials enrolling patients with paroxysmal AF, the overall estimate showed a significantly higher success rate for adjunctive GP or CFAE ablation (1.83 [1.25, 2.68]; P = 0.002), without significant heterogeneity for this outcome (I^2^ = 33%, P = 0.19). In this patient subgroup, adjunctive GP ablation demonstrated a significant treatment effect (2.53 [1.45, 4.42], P = 0.001), which was not the case for adjunctive CFAE ablation (1.37 [0.81, 2.32], P = 0.25) (Fig 5). Among the seven trials with non-paroxysmal AF, GP/CFAE + PVI ablation also showed significant treatment effect (1.40 [1.07, 1.83]; P = 0.01). Although both strategies demonstrated favorable results in subgroup analysis, the significant difference was not observed in adjunctive CFAE ablation (GP: 1.88 [1.14, 3.08], P = 0.01; CFAE: 1.24 [0.90, 1.71], P = 0.18,) (Fig 6).

### Freedom from arrhythmia after multiple procedures

Patients in 10 out of the 14 trials underwent repeat procedure. The rate of repeat ablation was 21% among patients who underwent adjunctive GP/CFAE ablation. One trial showed that 10% of patients who received PVI alone underwent repeat procedure with addition of the CFAE approach.

**Fig 7:**
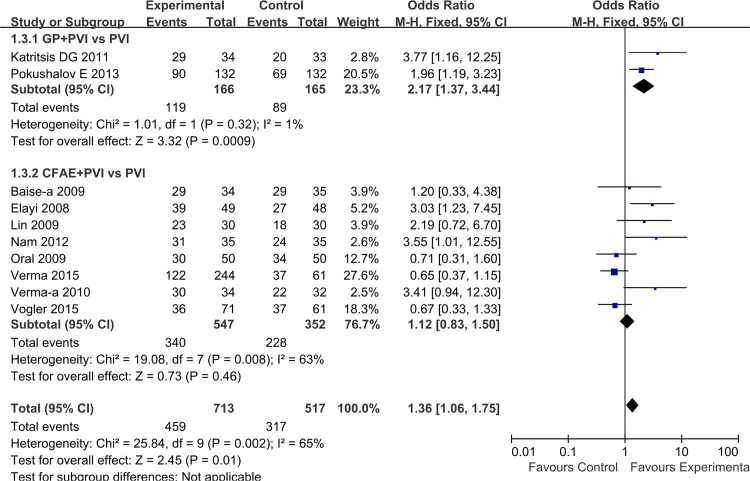
Effects of additional GP/CFAE ablation on sinus rhythm maintenance after repeat procedure.

In an overall estimate, compared with PVI alone, success rate of sinus rhythm maintenance after repeat procedure increased by additional GP/CFAE ablation (1.36 [1.06, 1.75]; P = 0.01), with significant heterogeneity for this outcome (I^2^ = 65%, P = 0.002). Subgroup analysis demonstrated that additional GP ablation significantly increased rate of freedom from AF/AT (2.17 [1.37, 3.44]; P = 0.0009), whereas adjunctive CFAE ablation improved the OR of maintaining sinus rhythm compared with PVI alone without significant difference (1.12 [0.83, 1.50]; P = 0.46) (Fig 7).

### Heterogeneous analysis

**Fig 8:**
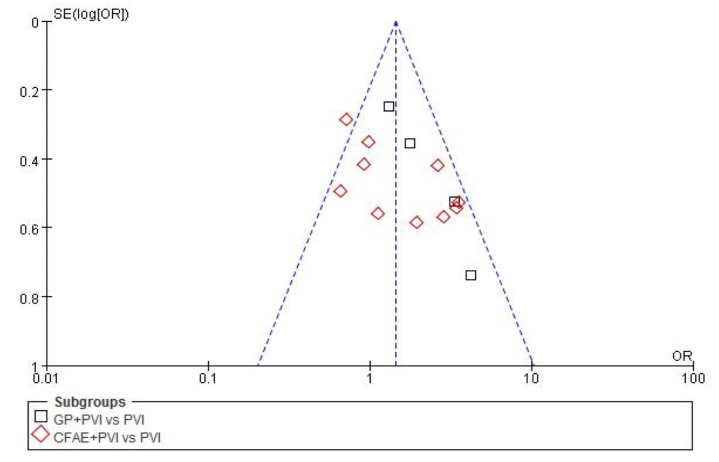
Funnel plot (with pseudo-95% confidence intervals) of studies in the meta-analysis.

A statistical analysis of funnel plots suggested publication bias in analysis of all 14 studies(I^2^ = 49%, P = 0.02)(Fig 8). A meta-regression analysis (Table 4) was performed to assess whether an interaction between the features of included studies, and incidence of AF recurrence after one or repeat procedure. In analysis of the 14 studies indicated that sample size (coefficient: -0.94, P<0.01) and ablation strategy (coefficient: -0.95, P<0.01) were significant sources of heterogeneity in outcomes after one procedure. For repeat procedure, only sample size of studies (coefficient: -1.39, P<0.01) related to heterogeneity.

**Table 4:** Meta-regression analysis.

	Coefficient	SE	95% CI	P value
***One procedure***				
Sample size (≥100 patients or less)	-0.94	0.31	-1.56 to -0.33	<0.01
Study design (RCT or non-RCT)	-0.65	0.43	-1.49 to 0.18	0.12
Ablation strategy (adjunctive CFAE or GP)	-0.95	0.32	-1.58 to -0.32	<0.01
Duration of follow-up (12 months or longer)	-0.46	0.29	-1.03 to 0.15	0.11
Type of AF (only paroxysmal or not)	-0.39	0.29	-0.98 to 0.19	0.12
***Repeat procedure***				
Sample size (≥100 patients or less)	-1.39	0.41	-2.19 to -0.60	<0.01
Study design (RCT or non-RCT)	0.26	1.17	-2.03 to 2.55	0.82
Ablation strategy (adjunctive CFAE or GP)	0.66	0.81	-0.92 to 2.24	0.41
Duration of follow-up (12 months or longer)	0.41	0.87	-1.29 to 2.11	0.63
Type of AF (only paroxysmal or not)	-0.33	0.56	-1.44 to 0.78	0.56

CI, confidence interval; SE, standard error.

## Discussion

### Main findings

In the present meta-analysis: 1) the addition of GP ablation to PVI significantly increased rate of sinus rhythm maintenance; 2) adjunctive CFAE ablation appeared not beneficial for AF, compared with PVI alone; and 3) compared with GP ablation, CFAE ablation was associated with higher rate of recurrence of AT or AFL.

### Adjunctive GP and CFAE ablation

In the present analysis, the combination of GP + PVI yielded better outcome than PVI alone, even for persistent AF. A prior meta-analysis also demonstrated that adjunctive cardiac autonomic denervation (CAD) is effective for both PAF and non-PAF[19,20]. However, the analysis of Zhou et al[19] included a low percentage (12%) of non-PAF. Another study concomitantly analyzed adjunctive GP and CFAE as CAD strategy for non-PAF [20]. In contrast to CFAE formation in PAF, a significant proportion of CFAE was caused by fibrosis-related anisotropic conduction and functional block in persistent AF. Thus, it is inappropriate to estimate the effects of adjunctive GP and CFAE as the CAD approach on sinus maintenance without desperation.

In the studies included in the present meta-analysis, anatomic GP ablation was used for paroxysmal AF instead of the selective approach relying on parasympathetic response elicited by high-frequency simulation (HFS). Compared with the anatomic approach, HFS-guided GP ablation is difficult to perform in patients not under general anesthesia, and may not elicit a vagal response at sites that are innervated by both sympathetic and parasympathetic nerves, leading to underestimation of the extent of the GP area[9,10]. Moreover, the CFAE was present more often at antral regions of PV in paroxysmal AF[21]. Therefore, extensive anatomic GP ablation may affect not only GP regions but also concomitantly ablate sites with CFAE in paroxysmal AF.

In sub-analysis of non-paroxysmal AF patients, only one study enrolled an adjunctive GP ablation group[9]: although more than 40% patients with long-standing AF were enrolled in this trial and the population had larger left atrial diameters, there was much improvement in outcome compared with PVI plus linear lesion. Notably, this proposed methodology for denervation does not represent a pure selective GP ablation approach because CFAE neighboring GP sites were also included in the target zone. In terms of the lesion region, it seems similar to that achieved with anatomic GP ablation.

In the present meta-analysis, adjunctive CFAE ablation did not provide benefit on clinical outcome in all sub-analyses. Of note, the STAR-AF II trial [6] contributed 30% weight to clinical outcome among ten studies. In this multicenter RCT, there was no reduction in the rate of recurrent AF associated with additional bi-atrial CFAE ablation. Furthermore, the results did not change after two procedures suggesting that extensive CFAE ablation might have been unnecessary. Although the definition of CFAE was based on an automated algorithm in all selected trials, the areas of CFAE ablation were not exactly the same. Overall, six trials included in the present analysis performed extensive CFAE ablation to left and right atria, and pooled analysis did not show improved clinical efficacy. A prior study demonstrated that further right atrial CFAE guided ablation offered an additional 50% AF termination[22], and had larger AT conversion rate before reaching final SR recovery. Although sufficient de-fragmentation at the right atria might reduce the critical mass necessary to maintain chronic bi-atrial AF, the role of right atrial CFAE remains incompletely elucidated, and more extensive ablation may cause new iatrogenic areas of arrhythmogenesis.

Previous studies and meta-analyses had focused on assessment of arrhythmia recurrence after ablation as clinical outcome; however, AT/AFL related to ablation account for approximately half of all recurrence events after extensive bi-atrial substrate ablation[23]. After adjunctive CFAE for AF ablation, Nadamanee et al reported that 36% of patients had subsequent AT/AFL, half with macro-reentrant circuits and half with focal mechanisms[24]. GP ablation is complicated by subsequent AT/AFL with a much lower incidence of 2%-10% [25]. Consistent with latter reports, the present analysis also showed that 16% and 4% of patients experienced AT/AFL after undergoing adjunctive CFAE and GP ablation, respectively. It stands to reason that the mechanisms of post-ablation AT are particularly variable in cases in which multiple strategies are used. Although in our experience not all these subsequent AT/AFL required a repeat procedure, the rather prominent proarrhythmic effect of CFAE ablation is a major limitation of this otherwise very effective strategy. The mechanism of these subsequent AT/AFL is typically macro-reentry and sometimes micro-reentry involving preexisting or iatrogenic ablation related scar tissue. Theoretically, the larger the region ablated, the higher the incidence of subsequent AT/AFL. Therefore, CFAE ablation was associated with more cases of subsequent AT/AFL than ablation at GP sites. There had been insufficient data to compare GP and CFAE ablation, and based on the present meta-analysis, the addition of anatomic GP ablation to PVI appears more promising than adjunctive CFAE ablation.

### Limitations

Firstly, because several studies had distinctly different AF populations and we did not have access to the original patient level data from each of the trials, pooling results for sub-analysis may underestimate the efficacy of ablation. Secondly, in Baise et al[15], a proportion of patients who received repeat procedures did not follow the initial randomization assignment regarding ablation strategy. Verma et al[17] had a small percentage of patients who remained on anti-arrhythmic drugs at follow-up. The latter features may affect the identification of outcome predictors. Thirdly, the total number of trials is relatively lower for the subgroup analysis of GP ablation. The reason for this limitation might be that the importance of neural mechanisms of AF is insufficiently recognized in many centers, and GP ablation as a more recent technique has not been widely used in the world. Future higher-quality and more rigorous randomized trials with longer follow-up on clinical effectiveness and safety of additional GP ablation are warranted.

## Conclusion

In the present meta-analysis, addition of GP ablation to PVI appeared to improve freedom from atrial arrhythmia, compared with PVI alone. However, addition of CFAE ablation to PVI appeared to confer no incremental clinical benefit in patients with paroxysmal or persistent AF, but rather to increase incidence of subsequent AT/AFL. The GP+PVI approach appeared more promising; however, further assessment in larger scale clinical trials for comparison between adjunctive GP and CFAE ablation strategies is warranted.

## Supporting Information

S1 Table Checklist items for present meta-analysis. (DOC)
